# The Effects of COVID-19 on the Placenta During Pregnancy

**DOI:** 10.3389/fimmu.2021.743022

**Published:** 2021-09-15

**Authors:** Habib Sadeghi Rad, Joan Röhl, Nataly Stylianou, Mark C. Allenby, Sajad Razavi Bazaz, Majid E. Warkiani, Fernando S. F. Guimaraes, Vicki L. Clifton, Arutha Kulasinghe

**Affiliations:** ^1^School of Biomedical Sciences, Queensland University of Technology, Brisbane, QLD, Australia; ^2^School of Chemical Engineering, University of Queensland, St Lucia, QLD, Australia; ^3^Centre for Biomedical Technologies, School of Mechanical, Medical and Process Engineering, Queensland University of Technology, Brisbane, QLD, Australia; ^4^School of Biomedical Engineering, University of Technology Sydney, Sydney, NSW, Australia; ^5^The University of Queensland Diamantina Institute (UQDI), Brisbane, QLD, Australia; ^6^Mater Research Institute, University of Queensland, Brisbane, QLD, Australia

**Keywords:** COVID-19, placenta, SARS-CoV-2, transplacental infection, pregnancy

## Abstract

Coronavirus disease 2019 (COVID-19) caused by the severe acute respiratory syndrome coronavirus 2 (SARS-CoV-2) has caused a global pandemic. The virus primarily affects the lungs where it induces respiratory distress syndrome ranging from mild to acute, however, there is a growing body of evidence supporting its negative effects on other system organs that also carry the ACE2 receptor, such as the placenta. The majority of newborns delivered from SARS-CoV-2 positive mothers test negative following delivery, suggesting that there are protective mechanisms within the placenta. There appears to be a higher incidence of pregnancy-related complications in SARS-CoV-2 positive mothers, such as miscarriage, restricted fetal growth, or still-birth. In this review, we discuss the pathobiology of COVID-19 maternal infection and the potential adverse effects associated with viral infection, and the possibility of transplacental transmission.

## Introduction

The World Health Organization (WHO) declared a global pandemic of coronavirus disease 2019 (COVID-19) in March 2020, caused by the Severe Acute Respiratory Syndrome Coronavirus 2 (SARS-CoV-2) (1). As of August 2021, the number of total cases surpassed 200 million and resulted in more than 4 million deaths. There is an ongoing effort to understand transmission, incidence, disease pathogenesis and the short- and long-term impacts following infection. In particular, the impact of SARS-CoV-2 infection on mothers and their babies (2). Evidence suggests that pregnant women with COVID-19 are more susceptible to severe disease with a higher risk of preterm birth (3–5), as well as higher risk of maternal and/or fetal death (6, 7). These findings are reminiscent of the dire outcomes from other similar respiratory viral infections, such as influenza A/H1N1 (8–11), severe acute respiratory syndrome (SARS) (12), and Middle East Respiratory Syndrome (MERS) (13, 14), where infected pregnant women are at increased risk of severe morbidity and mortality to both themselves and their infants (2). While most neonates born to SARS-CoV-2 positive mothers test negative and do not present with virus-induced disease, there have been some cases of newborns testing positive and presenting with early-onset symptoms (15). Whether this is due to the trans-placental transmission of SARS-CoV-2, or infection following delivery is still not well understood (16–18). Examination of the placentas from SARS-CoV-2 positive mothers have mixed reports on viral positivity, and not all neonates born from mothers with a SARS-CoV-2 positive placenta test positive for the virus (19). This suggests that there is a protective mechanism/barrier within the placenta, where its success may rely on the presence or absence of certain receptors/pathways. Fortunately, SARS-CoV-2 positive neonates are yet to present with any congenital defects (20). In this review, we provide an overview of the literature of SARS-CoV-2 infection during pregnancy, as well as the pathobiology of the placenta which may protect the growing fetus.

## Immune System Alterations During SARS-CoV-2 Infection

The immune system changes during pregnancy in such a way that it adapts to the growth of a semi-allogeneic fetus in the body of the mother, resulting in a distinct immune response to different infections during pregnancy (21–23). It has been well documented that in patients with COVID-19, particularly those with severe disease, have profound immune dysregulation (24). Studies have revealed an increase in blood leukocytes (leukocytosis), which was characterized by a decrease in lymphocytes (lymphopenia) and an increase in neutrophil-to-lymphocyte ratio (NLR) (25, 26). Using immunophenotyping analyses, researchers discovered that patients with severe COVID-19 had fewer natural killer (NK), CD3+, CD4+, and CD8+ T cells than those with the non-severe disease (27). NK cells were also found to be functionally exhausted during SARS-CoV-2 infection (28–30). Moreover, a reduction in circulating NK cell population has been reported during gestation (31). NK cells have key roles in the innate immune response by killing transformed cells, as consequence of viral infections or oncogenesis; NK cells are also major sources of pro-inflammatory cytokines such as granulocyte-macrophage colony-stimulating factor (GM-CSF) and interferon gamma (IFN-γ), which can restore or activate the antiviral property of the myeloid compartment; thus, any decrease in these cell populations may alter the ability to clear viruses (32). Evidence has shown that lymphopenia and enhanced NLR can be further amplified by COVID-19 disease severity (33). Compared to patients with moderate COVID-19, individuals with severe disease had lower numbers of cytotoxic T lymphocytes (CTLs) (33). Studies investigating COVID-19 patients’ lung tissue and bronchoalveolar lavage fluid (BALF) samples found T cell hyperactivation and/or upregulation of pro-apoptotic factors, including first apoptosis signal receptor (FAS), TNF‐related apoptosis‐inducing ligand (TRAIL), and caspase 3, as the main causes of T cell depletion (34–36). Alterations in CD4+ T cell population toward T helper-2 (Th)-2 phenotypes rather than Th1 phenotypes have been found during pregnancy, which contributes to the promotion of humoral immune responses over cellular immune responses (37). There is also a balance between regulatory T cell (Treg) and Th17 cells during pregnancy; with a shift towards Tregs to ensure fetal-maternal immune tolerance and to prevent fetal allograft rejection (38). In terms of innate immune cells, evidence suggests that, while absolute peripheral blood monocyte counts are not significantly different between patients with severe COVID-19 and those with moderate disease, the activation status of the monocyte/macrophage system is significantly altered (39). It was shown that monocyte/macrophage alterations caused by SARS-CoV-2 infection are similar to a condition known as familial hemophagocytic lymphohistiocytosis (HLH), a systemic inflammatory disorder involving cytokine production and cytopenia (40–42). HLH can be triggered either by abnormalities in genes regulating NK and cytotoxic CD8+ T cell degranulation or by conditions such as autoimmune disease, malignancy, and viral infection (40, 41). It was found that patients with H1N1 influenza who experienced the ‘cytokine storm,’ characterized by the extreme and excessive immune and inflammatory response (43), had mutations in genes associated with HLH (44). Many studies, however, do not support the link between HLH and COVID-19 (45–47). Wood et al., found that only three of 40 COVID-19 patients had Hscores >169, the cut-off used to identify HLH (47). Several studies have reported widespread infiltration of monocytes/macrophages in the lung tissue samples taken from COVID-19 patients (35, 48, 49). Single-cell studies revealed that monocyte-derived FCN1+ macrophages were the most abundant macrophage subset found in BALF samples from severe COVID-19 patients (35). Furthermore, it was discovered that peripheral monocyte trafficking and subsequent differentiation into macrophages in the lungs of COVID-19 patients contributes to pro-inflammatory responses and further activation of innate immune cells (49). Changes in the innate immune system during pregnancy, also, involve the pattern recognition receptors Toll-like receptors (TLRs), in particular TLR4 (50, 51). There are three different levels of TLR4 activation during pregnancy. First, TLR4 activation and the inflammatory response rise during the first trimester, allowing blastocyst implantation. Following that, a decrease in TLR4 activation happens during the second trimester in order to create an anti-inflammatory response for fetal growth. Eventually, TLR4 activation and the inflammatory response increase again in the third trimester to support labor and delivery (52). Infection with COVID-19 leads to pyroptosis of host cells and the release of danger associated molecular patterns (DAMPs) that can act as ligands for TLR molecules and trigger a greater inflammatory response (31). Studies are needed to determine whether such changes in the immune system result in higher susceptibility or are protective against COVID-19 during pregnancy (31).

### Expression of ACE2 and TMPRSS2 in Placental and Fetal Cells

**Figure 1:**
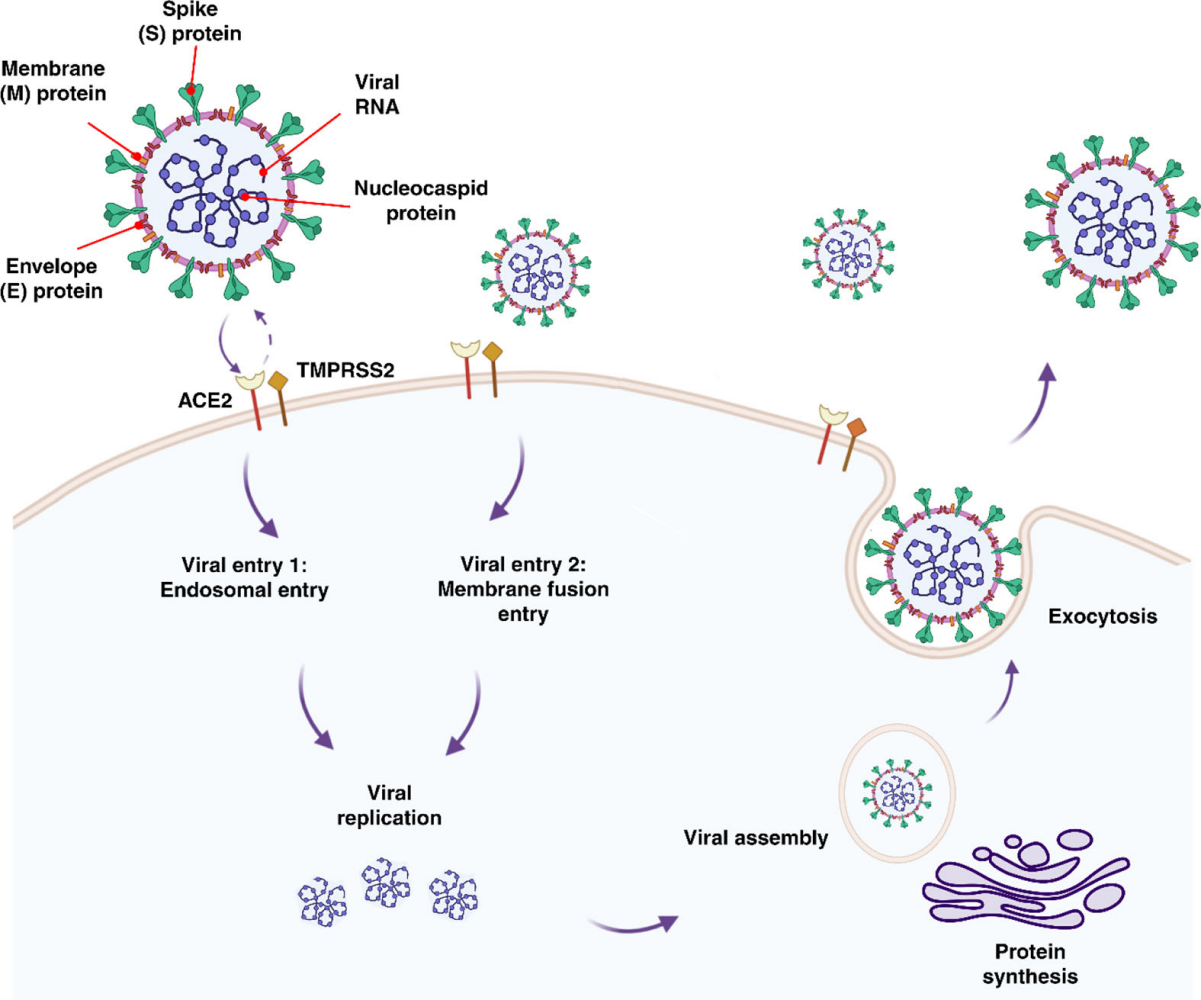
Features, entry methods, and replication of SARS-CoV-2.

SARS-CoV-2 enters the body through the nasal passage and infects pulmonary cells by binding to the receptor angiotensin-converting enzyme 2 (ACE2) (31, 53–55). It has been found that ACE2 expresses in respiratory and intestinal track, placenta, ovaries, vagina, and uterus (56). Cell entry is further facilitated by viral spike (S) protein priming induced by trans-membrane serine protease 2 (TMPRSS2) (53–55). Cells co-expressing both ACE2 and TMPRSS2 have been found to have a higher susceptibility to SARS-CoV-2 entry (57) (**Figure 1**). In addition, furin, trypsin, and cathepsins B and L have been reported to be capable of cleaving the spike glycoprotein binding at the S1/S2 site, allowing the virus to enter (53, 58, 59). ACE2 has been shown to be expressed by fetal kidney, ilium, and rectal cells from as early as 15 weeks, barely detectable at 15 weeks in the lungs with undetected expression thereafter, and undetectable in the cerebral ependymal, parenchymal and cardiac cells (60). It has been found that only a proportion of cells which are located in the fetal adrenal gland and the kidney co-expressed ACE2 and TMPRSS2. It was discovered that placental cytotrophoblasts and syncytiotrophoblasts (STBs) express ACE2 from 7 weeks onward, suggesting that SARS-CoV-2 could cross into the placenta at any gestational age (60). Investigation of ACE2 and TMPRSS2 co-expression in the developing embryo up to day 14 (from surplus IVF human embryos) has revealed the co-localization of these genes, raising concern to increased susceptibility to SARS-CoV-2 fetal infection in the early stages of embryonic development (61). To date, cohort studies of SARS-CoV-2 positive mothers with mild symptoms or asymptomatic, have reported no adverse effects to the mother or neonate regardless of the timing of the infection (i.e. first versus third trimester) (62, 63). However, women with severe SARS-CoV-2 infection that required critical care had higher odds of complications, particularly a higher incidence of iatrogenic pre-term delivery mostly due to fear of sudden maternal decompensation (64).

## Transplacental Viral Transmission

**Figure 2:**
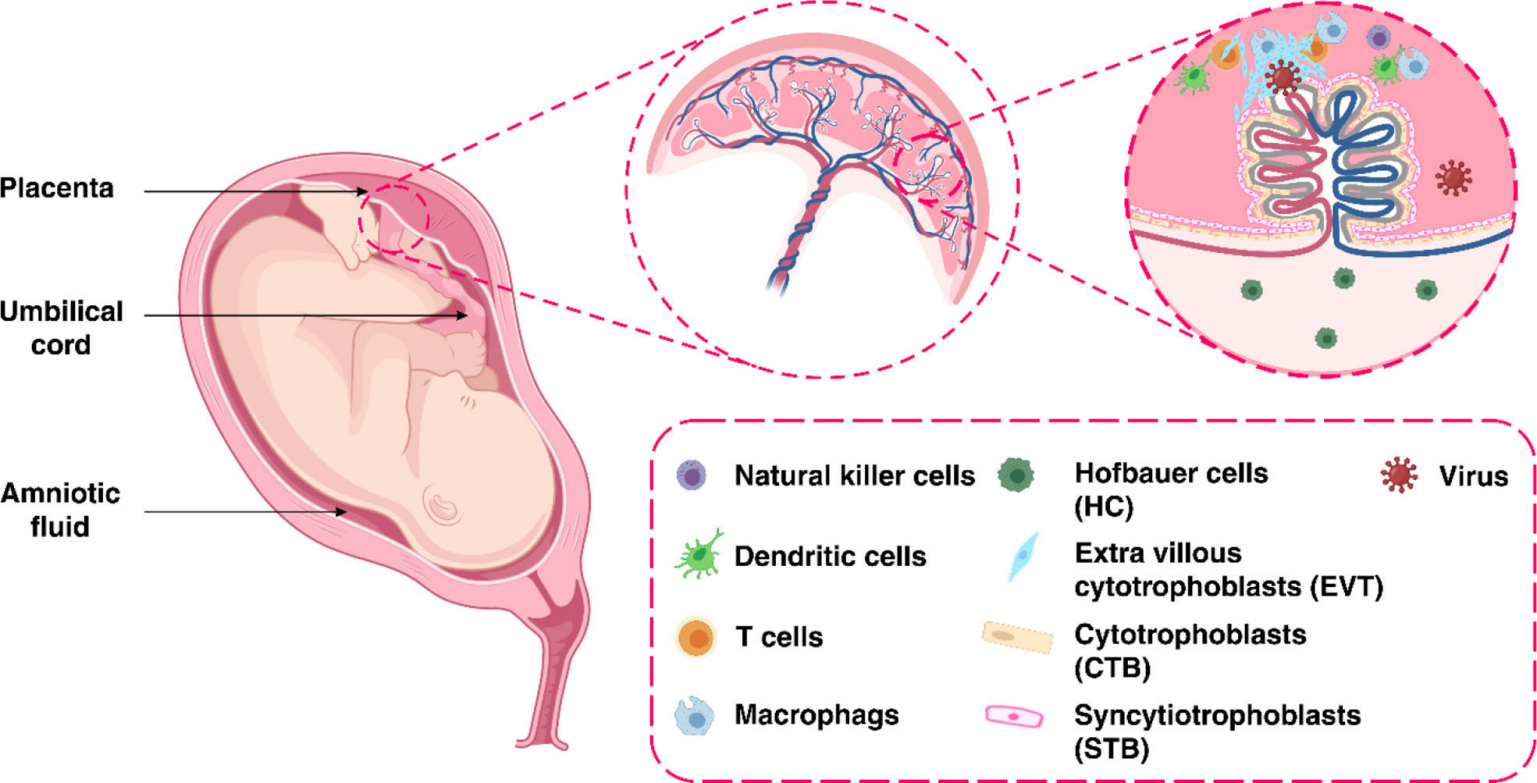
The maternal-fetal interface.

**Figure 3:**
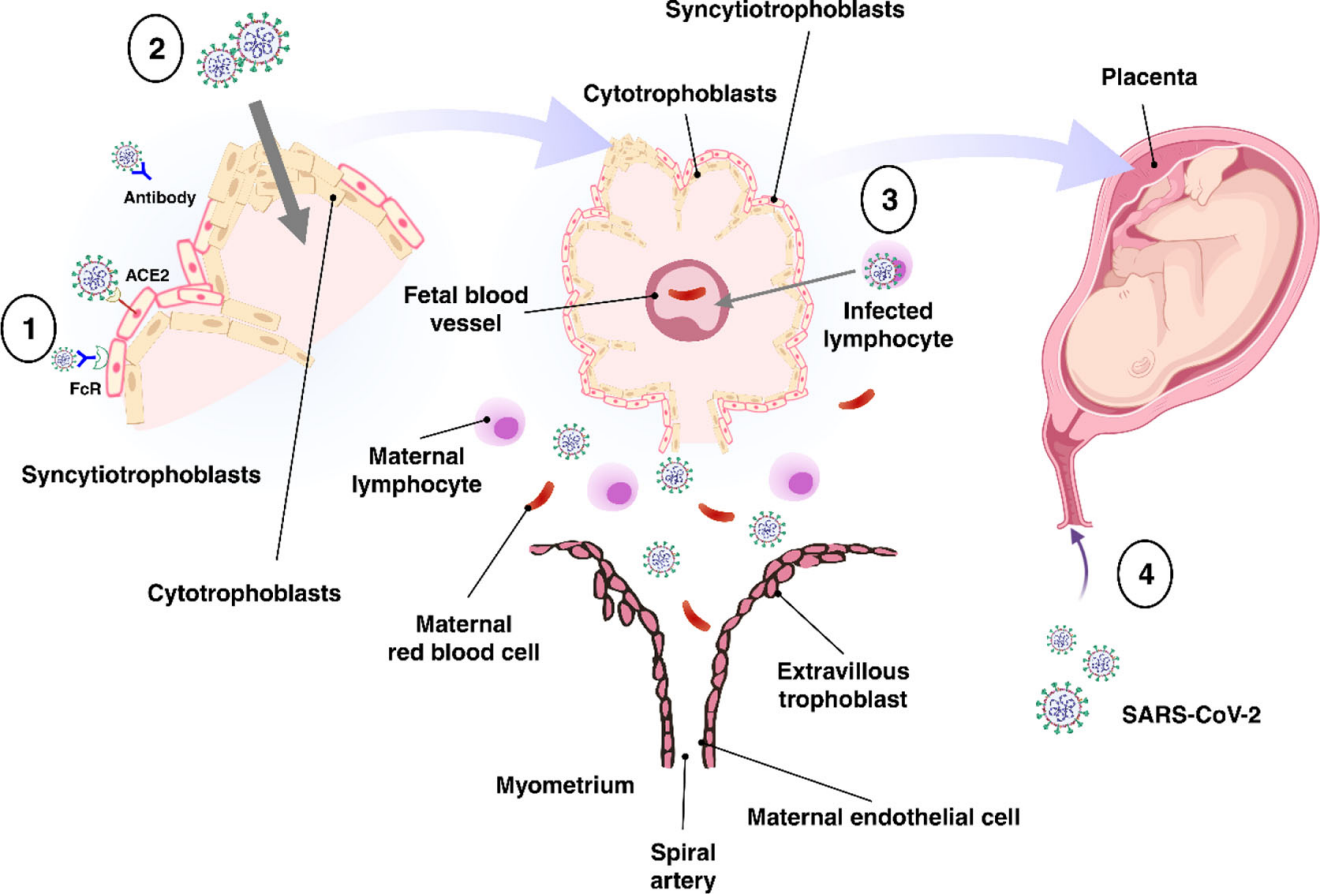
Possible mechanisms of transplacental transmission. There are several potential mechanisms involved in the virus**’**s vertical transmission from mother to fetus. (1) Infection caused by direct villous tree damage. (2) Infection through the maternal endothelium to the extravillous trophoblast. (3) Infection caused by maternal immune cell trafficking and transcellular transport. (4) Infection through the vagina. Adapted from (67).

The placenta offers a protective barrier that does not allow the fetus to become exposed to maternal infections (31). The human placenta primarily consists of a number of specific fetal-derived cells called trophoblasts, of which there are three main types. These include terminally differentiated multinuclear syncytiotrophoblast cells, which are in direct contact with the maternal blood and line the villus tree, progenitor villous cytotrophoblast cells, which underlie the syncytiotrophoblast, and invasive extravillous trophoblast (EVT) cells, which anchor the chorionic villi to the uterus and modify its vasculature (**Figure 2**) (31). Various potential causes may play a role in the vertical transmission of the virus from the mother to the fetus. These include direct damage to the villous tree with a break in the protective syncytiotrophoblast layer, which could be caused by virus-induced apoptosis and vascular damage in the placenta, spread through the virus-infected maternal endothelium to the extravillous trophoblast, trafficking of infected maternal immune cells throughout the syncytiotrophoblast, paracellular or transcellular transport (for example, immunoglobulin-mediated transcytosis) into fetal capillaries, transmission *via* swallowed or aspirated amniotic fluid (65, 66), as well as ascending infection from the vagina (**Figure 3**) (31). To define the possibility of vertical transmission of SARS-CoV-2 infection in different studies, a classification system has been proposed by a multidisciplinary team of the WHO (68). Given the timing of vertical transmission, *in utero*, intrapartum, and early postnatal period, four possibilities exist: confirmed, possible, unlikely, and indeterminate (68). Vertical transmission is considered “possible” if evidence suggests it but cannot confirm infection. However, if there is poor support of diagnosis, but vertical transmission cannot be completely ruled out, this is considered as “unlikely”. The “indeterminate” possibility is when the tests required to define the classification have not been performed (68). Recent findings confirming the presence of SARS-CoV-2 mRNA or virions in syncytiotrophoblasts have strongly suggested transplacental infection caused by the SARS-CoV-2 (69, 70). Nonetheless, given that the presence of SARS-CoV-2 in the blood sample of COVID-19 patients is reported to be around 1%, therefore the likelihood of SARS-CoV-2 being able to directly infect syncytiotrophoblasts is low (71). Another alternative way of transmitting SARS-CoV-2 infection to the neonate is through the vagina during childbirth (72, 73).

Whilst the possibility of transmitting SARS-CoV-2 from mother to fetus during pregnancy is suggested, the role of the placenta in infection with the virus has not yet been fully understood. However, evidence suggests that pathogens can overcome this barrier, infect the fetus, and even cause serious complications in newborns, such as microcephaly and ocular abnormalities (74). Such pathogens include Cytomegalovirus (CMV), herpes simplex virus (HSV), varicella-zoster virus, and Zika virus (ZIKV) (20, 75–77). It is currently unclear whether neonates who tested positive for SARS-CoV-2 have been infected with the virus from their mothers during pregnancy or have been infected during labor or after birth. (**Table 1**). Evidence based on infant antibody tests suggests vertical transmission of the virus may be possible. It was discovered that infants born to women infected with SARS-CoV-2 had higher immunoglobulin (Ig)G and IgM levels for SARS-CoV-2 (31, 89, 90). The presence of IgG in the fetus may indicate the transfer of this immunoglobulin from the mother to the fetus during pregnancy, but the presence of IgM indicates that the fetus has produced and secreted this immunoglobulin in response to viral infection because in contrast to IgG, IgM is unable to cross the placenta due to its higher molecular weight (89, 90).

**Table 1:** Systematic review and meta-analysis studies on COVID-19 infection during pregnancy.

Publication name	Number of pregnant women with COVID-19	Findings	Conclusion
Vertical transmission of coronavirus disease 2019: a systematic review and meta-analysis (78)	NM	SARS-CoV-2 RNA positivity was as follows 0% (0/51) in amniotic fluid 0% (0/17) in urine 3.6% (1/28) in the cord blood 7.7% (2/26) by placental sample analysis 9.7% (3/31) by rectal or anal swab	Vertical transmission of SARS-CoV-2 is possible but the likelihood of its occurrence is low The rate of SARS-CoV-2 infection is almost similar to other pathogens causing congenital infections
Clinical outcomes of 201 neonates born to mothers with COVID-19: a systematic review (79)	223	Fetal death was reported in two cases Preterm birth was reported in 48 of 185 newborns Birth asphyxia was reported in 1.8% of neonates Respiratory distress syndrome was reported in 6.4% of neonates	SARS-CoV-2 infection during pregnancy rarely affects fetal and neonatal mortality SARS-CoV-2 infection during pregnancy can affect the fetal and neonatal morbidity
Maternal clinical characteristics and perinatal outcomes among pregnant women with coronavirus disease 2019. A systematic review (80)	322	Premature birth was reported as the main adverse obstetric outcome in pregnant women SARS-CoV-2 infection was not reported in samples, including breast milk, amniotic fluid, placenta or umbilical cord blood	The study did not support the possibility of vertical transmission of SARS-CoV-2 in the third trimester
Clinical characteristics and outcomes of pregnant women with COVID-19 and the risk of vertical transmission: a systematic review (81)	230	Premature birth was reported in 24.74% (24 out of 97) of newborns SARS-CoV-2 infection was not reported in samples, including vaginal secretions, breast milk, amniotic fluid, placental blood, and placental tissues 3.9% (5 out of 128) of newborns tested positive for SARS-CoV-2 RNA	The main adverse event for newborn was premature delivery
Clinical characteristics and outcomes of pregnant women with COVID-19 and comparison with control patients: A systematic review and meta-analysis (82)	10,000	Preterm birth was more common in pregnant women with COVID-19 than pregnant women without COVID-19 The rate of vertical transmission was 5.3% The rate of SARS-CoV-2 infection in neonates born to mothers with COVID-19 was 8%	The higher likelihood of preterm birth in pregnant women with COVID-19 compared to pregnant women without COVID-19 may suggest a possible link between COVID-19 infection and pregnancy complications
Clinical Characteristics and Neonatal Outcomes of Pregnant Patients With COVID-19: A Systematic Review (83)	235	SARS-CoV-2 infection was not reported in samples, including breast milk, amniotic fluid, and neonatal throat swab Preeclampsia and premature delivery were reported as the major complications in pregnant women with COVID-19	The study did not support the possibility of vertical transmission of SARS-CoV-2 infection, however it mentioned that the vertical transmission cannot be ignored
Pregnancy and Breastfeeding During COVID-19 Pandemic: A Systematic Review of Published Pregnancy Cases (84)	3,985	Preterm birth was recorder in 23% of cases SARS-CoV-2 infection was reported in samples, including amniotic fluid, breast milk, placenta, and cord blood, from pregnant women with COVID-19 61 newborns were found to be tested positive for SARS-CoV-2	The study suggested that vertical transmission of SARS-CoV-2 is possible
COVID-19 (SARS-CoV-2) Infection in Pregnancy: A Systematic Review (85)	156	Intrauterine/fetal distress and premature rupture of membranes were reported as the most common maternal/fetal complications	The study suggested that COVID-19 infection may increase the risk of preterm birth and maternal death The study did not support the possibility of vertical transmission of SARS-CoV-2 infection
Maternal and perinatal outcomes with COVID-19: A systematic review of 108 pregnancies (17)	108	Maternal intensive care unit (ICU) admission was reported One case of intrauterine fetal death and one case of neonatal case was reported	The study mentioned that the vertical transmission cannot be ruled out
COVID-19 in Pregnant Women and Neonates: A Systematic Review of the Literature with Quality Assessment of the Studies (86)	275	Preterm birth was recorded in 28% of cases 2 stillbirths were reported 16 out of 248 neonates were tested positive for SARS-CoV-2 RNA, of which 9 of them were born to mothers with COVID-19 SARS-CoV-2 infection was not reported in samples, including amniotic fluid, vaginal/cervical fluids, breast milk, and placental tissue	The study mentioned that the vertical transmission is unlikely but it cannot be ruled out
Maternal Coronavirus Infections and Neonates Born to Mothers with SARS-CoV-2: A Systematic Review (87)	1457	64 cases of premature birth were reported 16 cases of intrauterine fetal death or neonatal death were reported 15 cases of maternal death were reported 7 cases of miscarriage were reported 19 cases of decreased fetal movements were reported 5 cases of severe neonatal asphyxia were reported 39 out of 1042 newborns were tested positive for SARS-CoV-2 infection SARS-CoV-2 infection was reported in samples, including breast milk and placenta	The study suggested that COVID-19 infection can be associated with maternal, fetal, and neonatal complications The study mentioned that the vertical transmission cannot be ruled out
Vertical transmission of SARS CoV-2: a systematic review (88)	714	17 out of 606 neonates were tested positive for SARS-CoV-2 RNA SARS-CoV-2 infection was reported in samples, including amniotic fluid, placenta and breast milk	Possible vertical transmission of SARS-CoV-2 has been reported in some studies

NM, not mentioned; SARS-CoV-2, Severe Acute Respiratory Syndrome Coronavirus 2; COVID-19, coronavirus disease 2019.

## Biomarkers of SARS-CoV-2 Infection

Several studies have employed single cell RNA sequencing (scRNA-seq) to gain an understanding of the molecular features of SARS-CoV-2 infection (91–95). In a study by Lu et al., which compared ACE2 and TMPRSS2 gene expression between fetal, placental tissues and adult tissues, a small proportion of trophoblast cells, as well as various fetal organs such as the heart, kidney, stomach, and adrenal glands, had ACE2 expression. The study showed that only the kidney and adrenal gland expressed TMPRSS2 (96). Pique-Regi et al. discovered that very few cells during any of the three trimesters expressed both ACE2 and TMPRSS2. Using single-nuclear RNAseq (snRNA-seq), it has been shown that the placenta is unlikely to express ACE2 and TMPRSS2, and thus be infected by SARS-CoV-2 (59). Using scRNA-seq data, Ashary et al., identified only a small proportion of STB in the first trimester and EVT in the second trimester had ACE2 and TMPRSS2 expression. The ACE2^+^TMPRSS2^+^STBs were highly differentiated and expressed genes engaged in mitochondrial metabolism and glucose transport. In addition, the ACE2^+^TMPRSS2^+^EVTs were found to have endovascular trophoblast markers. The researchers found that these cells could be the targets of SARS-CoV-2 entry (97). Moreover, robust immune responses at the maternal-fetal interface of SARS-CoV-2-infected women was discovered (98). Researchers found overexpression of interferon-related genes, and increased activation of NK cells and T cells (98–100). Also, it was found that there was an association between SARS-CoV-2 infection and local immune responses at the maternal-fetal interface (98). in a study by Nagy et al, the impact of mutations in SARS-CoV-2 viral genes on clinical outcomes was explored. The study found that mutations in the nucleocapsid phosphoprotein-N, nonstructural proteins-4 (NSP4), NSP6, Open Reading Frame-3a (ORF3a), and ORF8 were associated with mild outcome, while mutations in NSP7 were linked to severe disease (101).

The identification of new biomarkers and prevention strategies requires the fundamental understanding and control of how SARS-CoV-2 spreads to the lungs and elicits a multi-organ inflammatory response. (**Table 2**). These infection processes rely on their location and spatial context: which cells in which tissue locations are most susceptible to infection (105), infected cell-to-uninfected cell associations, and biochemical factor release of different cell types in response to infection (106). These spatiotemporal relationships in the inflammatory cascade give rise to positive or negative prognoses, and their understanding can triage patients at greater or lesser risks of infection, of response to infection, and inform new therapeutics and treatment regimens (107). Spatial immunoprofiling is rapidly advancing due to several recent technologies: advanced instrumentation, molecular barcoding and immunolabelling, providing a much richer portrait of the immune landscape (108), and recent approaches in biostatics and theoretical biology are incorporating imaging data to deconstruct the relationships between cells and disease within their tissue context (109–111). Spatial resolved transcriptomics are changing the ways in which we interrogate complex tissues and were voted the ‘Method of the Year 2020’ by the journal Nature Methods (112). These technologies combine the benefits in advancements in microscopy and advanced imaging, with simultaneous read out of transcript and proteomic data, thereby alleviating the challenges associated with single cell or bulk profiling. The maintenance of spatial context is key in understanding the underlying cellular profiles, biology, specialization and tissue organization and has begun shedding light into consortia studies such as the Human Cell Atlas. A number of technologies currently exist for RNA applications: Nanostring GeoMX Digital Spatial Profiler (DSP), 10x Genomics Visium, MERFISH and proteomic: Nanostring GeoMX DSP, Akoya Biosciences CODEX, Imaging Mass spectrometry (IMC) (113). Recent application of these methodologies to COVID-19 autopsy tissue studies from lungs, kidney, liver and heart tissue has provided deep insights into cell types and genes implicated with severe COVID-19 disease severity (114).

**Table 2:** Potential biomarkers of disease severity in COVID-19.

Analytes	Changes	Role	Ref
IL-1, IL-2, IL-6, TNF-a, G-CSF, GM-CSF, IFN-γ	Increase	Cytokine storm biomarker	(102)
CD3+, CD4+, CD8+, B cells, NK cells	Decrease	Clinical Hematological biomarker	(103)
CK, CK-MB, CRP, Ferritin, LDH, BUN, Creatinine, cTnI, AST, ALT, Total bilirubin	Increase	Clinical Biochemical biomarker	(104)

IL, interleukin; TNF-a, tumor necrosis factor; GM-CSF, granulocyte-macrophage colony-stimulating factor; G-CSF, granulocyte colony-stimulating factor; IFN-γ, interferon gamma; NK, natural killer; CK, creatine kinase; LDH, lactate dehydrogenase; BUN, blood urea nitrogen; cTnI, cardiac troponin I; AST, aspartate aminotransferase; ALT, alanine aminotransferase.

Once region- or cell-specific spatial information is derived from histology sections, statistical relationships between cells and tissues and mathematical predictions of their future behavior with or without treatment are often sought. There exist numerous tools to detect and segment single cell locations from this spatial information. While open-source *ImageJ*, developed in 1987, remains popular for microscopic image analysis (115–117), more recent software such as *CellProfiler*, *Icy*, *ilastik*, and *QuPath* provide user-friendly interfaces for the development of bioimage analysis macroscripts (118, 119). Once single-cell data can be derived, spatial relationships can be determined. The most common of which is intercellular clustering or associations, often calculated as cell density within concentric circles away from each cell’s center and averaged across all imaged cells (120). For instance, to characterize the distribution of SARS-CoV-2 bodies from macrophages or monocytes or tissue structures to estimate inflammatory progression (121).

## Concluding Remarks and Future Perspectives

Taking into account the changing physiology and immune responses during gestation, pregnant women are more susceptible to developing severe COVID-19, which can lead to pregnancy-related complications. There is limited information for the association of COVID-19 and its direct complications to the growing fetus during pregnancy. These may include preterm birth, stillbirth, or long-term complications for the newborn (122). A study conducted on 827 pregnant women, who have been given the COVID-19 mRNA vaccine, found that the proportion of adverse pregnancy and neonatal outcomes were similar to incidence reported in similar studies conducted prior to the pandemic (123). Furthermore, vaccination of pregnant women has been shown to result in maternal IgG production 5 days after the first dose of vaccination, as well as the transplacental transfer of IgG 16 days after the first dose of vaccination (124). However, longitudinal follow-up is needed to monitor those who are vaccinated, especially during the first trimester, in order to be informed about maternal, pregnancy, and neonatal outcomes. Another important consideration with COVID-19 infection during pregnancy is that current diagnostic tests such as X-ray and CT scans cannot be performed in pregnant women due to potential risks to the growing fetus (125). These factors may therefore delay the diagnosis and treatment of pregnant women, particularly those with more severe symptoms.

These factors may therefore delay the diagnosis and treatment of pregnant women, particularly those with more severe symptoms. Screening tests may be helpful in this respect because of the possibility of transmitting the virus from the mother to the fetus. Understanding the disease progression and its relationship to manifestation severity is necessary to therapeutically intervene and reduce the associated morbidity.

## Author Contributions

All authors listed have made a substantial, direct, and intellectual contribution to the work, and approved it for publication.

## Funding

This project is supported by the Queensland University of Technology (QUT) ECR grant to AK, MA, NS, and JR. NS is supported by a US Department of Defense Prostate Cancer Early Investigator Award Fellowship (PC190533). MA is supported by an Advance Queensland Fellowship (AQIRF1312018). FSFG is funded by a UQ Diamantina Institute laboratory start-up package, Australian and New Zealand Sarcoma Association – Sarcoma Research Grant, a priority-driven collaborative cancer research scheme grant co-funded by Cancer Australia and Cure Cancer (#1158085), and a US Department of Defense – Breast Cancer Research Program – breakthrough award level 1 (#BC200025). AK is supported by an NHMRC Fellowship (APP1157741) and Cure Cancer (APP1182179).

## Conflict of Interest

FSFG is a consultant and has a funded research agreement with Biotheus Inc.

The remaining authors declare that the research was conducted in the absence of any commercial or financial relationships that could be construed as a potential conflict of interest.

## Publisher’s Note

All claims expressed in this article are solely those of the authors and do not necessarily represent those of their affiliated organizations, or those of the publisher, the editors and the reviewers. Any product that may be evaluated in this article, or claim that may be made by its manufacturer, is not guaranteed or endorsed by the publisher.
