# Corrigendum: The effects of COVID-19 on the placenta during pregnancy

**DOI:** 10.3389/fimmu.2022.998406

**Published:** 2022-09-08

**Authors:** Habib Sadeghi Rad, Joan Röhl, Nataly Stylianou, Mark C. Allenby, Sajad Razavi Bazaz, Majid E. Warkiani, Fernando S. F. Guimaraes, Vicki L. Clifton, Arutha Kulasinghe

**Affiliations:** ^1^School of Biomedical Sciences, Queensland University of Technology, Brisbane, QLD, Australia; ^2^School of Chemical Engineering, University of Queensland, St Lucia, QLD, Australia; ^3^Centre for Biomedical Technologies, School of Mechanical, Medical and Process Engineering, Queensland University of Technology, Brisbane, QLD, Australia; ^4^School of Biomedical Engineering, University of Technology Sydney, Sydney, NSW, Australia; ^5^The University of Queensland Diamantina Institute (UQDI), Brisbane, QLD, Australia; ^6^Mater Research Institute, University of Queensland, Brisbane, QLD, Australia

**Keywords:** COVID-19, placenta, SARS-CoV-2, transplacental infection, pregnancy

In the published article (31) was not cited in the article. The citation has now been inserted in **Transplacental Viral Transmission**, paragraph two, the corrected paragraph appears below.

“Whilst the possibility of transmitting SARS-CoV-2 from mother to fetus during pregnancy is suggested, the role of the placenta in infection with the virus has not yet been fully understood. However, evidence suggests that pathogens can overcome this barrier, infect the fetus, and even cause serious complications in newborns, such as microcephaly and ocular abnormalities (73). Such pathogens include Cytomegalovirus (CMV), herpes simplex virus (HSV), varicella-zoster virus, and Zika virus (ZIKV) (20, 74–76). It is currently unclear whether neonates who tested positive for SARS-CoV-2 have been infected with the virus from their mothers during pregnancy or have been infected during labor or after birth. (Table 1). Evidence based on infant antibody tests suggests vertical transmission of the virus may be possible. It was discovered that infants born to women infected with SARS-CoV-2 had higher immunoglobulin (Ig) G and IgM levels for SARS-CoV-2 (31, 88, 89). The presence of IgG in the fetus may indicate the transfer of this immunoglobulin from the mother to the fetus during pregnancy, but the presence of IgM indicates that the fetus has produced and secreted this immunoglobulin in response to viral infection because in contrast to IgG, IgM is unable to cross the placenta due to its higher molecular weight (88, 89).”

Additionally, in the published article Komine-Aizawa S, Takada K, Hayakawa S. Placental barrier against COVID-19. Placenta. 2020 Sep 15;99:45-49. doi: 10.1016/j.placenta.2020.07.022 was not cited. The citation has now been inserted in the **Figure 3 **legend as shown and the corresponding reference added to the reference list.

**Figure 3:**
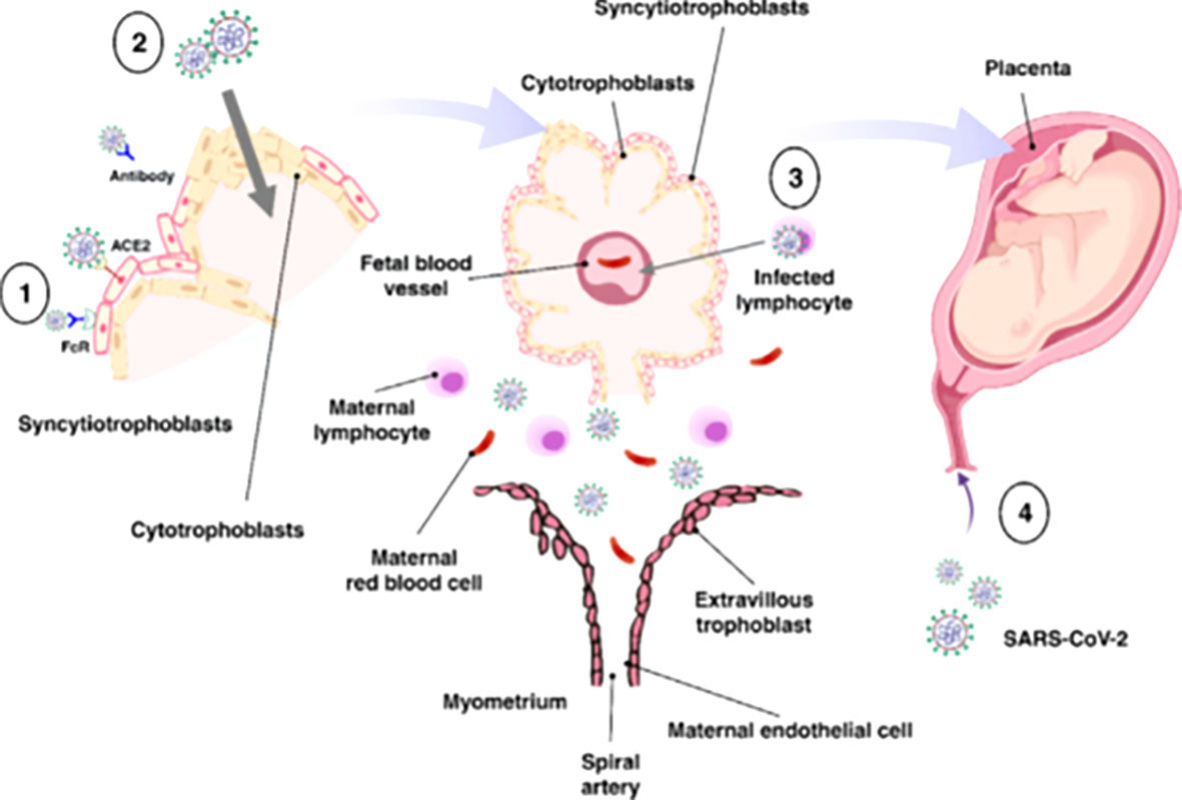
Possible mechanisms of transplacental transmission. There are several potential mechanisms involved in the virus’s vertical transmission from mother to fetus. (1) Infection caused by direct villous tree damage. (2) Infection through the maternal endothelium to the extravillous trophoblast. (3) Infection caused by maternal immune cell trafficking and transcellular transport. (4) Infection through the vagina. Adapted from (67).

The authors apologize for these errors and state that this does not change the scientific conclusions of the article in any way.

## Conflict of interest

The authors declare that the research was conducted in the absence of any commercial or financial relationships that could be construed as a potential conflict of interest.

## Publisher’s note

All claims expressed in this article are solely those of the authors and do not necessarily represent those of their affiliated organizations, or those of the publisher, the editors and the reviewers. Any product that may be evaluated in this article, or claim that may be made by its manufacturer, is not guaranteed or endorsed by the publisher.
